# An *n*-Dimensional Chaotic Map with Application in Reversible Data Hiding for Medical Images

**DOI:** 10.3390/e26030254

**Published:** 2024-03-13

**Authors:** Yuli Yang, Ruiyun Chang, Xiufang Feng, Peizhen Li, Yongle Chen, Hao Zhang

**Affiliations:** 1 College of Computer Science and Technology, Taiyuan University of Technology, Jinzhong 030600, China; yangyuliyyl@126.com (Y.Y.); chenyongle@tyut.edu.cn (Y.C.); 2 College of Software, Taiyuan University of Technology, Jinzhong 030600, China; changruiyun0927@126.com (R.C.); feng_tyut@126.com (X.F.); 19135733503@163.com (P.L.)

**Keywords:** chaotic map, reversible data hiding, privacy protection, secure communication

## Abstract

The drawbacks of a one-dimensional chaotic map are its straightforward structure, abrupt intervals, and ease of signal prediction. Richer performance and a more complicated structure are required for multidimensional chaotic mapping. To address the shortcomings of current chaotic systems, an *n*-dimensional cosine-transform-based chaotic system (*n*D-CTBCS) with a chaotic coupling model is suggested in this study. To create chaotic maps of any desired dimension, *n*D-CTBCS can take advantage of already-existing 1D chaotic maps as seed chaotic maps. Three two-dimensional chaotic maps are provided as examples to illustrate the impact. The findings of the evaluation and experiments demonstrate that the newly created chaotic maps function better, have broader chaotic intervals, and display hyperchaotic behavior. To further demonstrate the practicability of *n*D-CTBCS, a reversible data hiding scheme is proposed for the secure communication of medical images. The experimental results show that the proposed method has higher security than the existing methods.

## 1. Introduction

In recent years, nonlinear theory has received more and more attention. As a typical branch of nonlinear theory, chaos theory has been widely used in mathematics, medicine, physics, computer science, astronomy, ecology, and other scientific and engineering fields since its emergence [1]. A nonlinear system exhibiting chaotic behavior should possess high sensitivity, ergodicity, unpredictability, and initial value sensitivity, as per Devaney’s definition [2]. These important properties make it popular in security applications such as audio encryption [3,4], image encryption [5,6,7], image watermarking [8,9], and data hiding [10,11,12]. For these chaotic-based applications, the security largely depends on the performance of the underlying chaotic system.

### 1.1. Chaotic Systems-Related Work

Based on the number of state variables involved, chaotic systems can be loosely classified as one-dimensional chaotic maps or n-dimensional chaotic maps. Simple structure, low computing cost, and convenient hardware are some of the benefits of 1D chaotic mapping. Nevertheless, these benefits are sometimes accompanied by a lack of security. Many one-dimensional chaotic maps exhibit discontinuous chaotic ranges [13], which can cause chaotic behavior to degenerate into regular behavior. Motivated by the shortcomings of current one-dimensional chaotic maps, scientists have started working to enhance one-dimensional chaotic maps’ chaotic performance. Hua et al. [14] coupled two one-dimensional chaotic maps by cosine transform and constructed a one-dimensional cosine chaotic map intensifier. Li et al. [15] built a one-dimensional exponential chaotic map enhancer, increased the input chaotic map’s randomness, and added exponential components to the chaotic system structure. Hu et al. [16] designed a new chaos model UCS, which improved the statistical properties of existing maps and expanded the parameter range by concatenating two 1D chaotic maps and performing modular operations. Mansouri et al. [17] proposed a novel one-dimensional chaotic mapping amplifier (1-DCMA), which enhances the sensitivity of the input one-dimensional chaotic mapping through cosine and logarithm operations.

### 1.2. Data Hiding-Related Work

Data hiding (DH), or data embedding, is a method that allows secret data to be correctly extracted from an original media while preventing any visible distortions. Based on this, the reversible data hiding (RDH) technique is refined, which can both restore the original coverage media losslessly and extract the embedded data without error. These qualities make data concealment a crucial component of many multimedia systems. Prasad et al. [18] designed a steganographic scheme for RGB color images based on a binary lower triangular matrix, which reduces aberrations on steganographic images while maintaining significant visual quality. A non-sensing medical data-hiding system based on multi-resolution singular value decomposition, redundant discrete wavelet transform (RDWT), and non-subsampled shear wave transform (NSST) was created by Anand et al. [19]. The security of the concealment mechanism is guaranteed by the key-based encryption scheme. Based on the Chinese residue theorem, Yu et al. [20] present a new method for hybrid encoding and secret sharing. In particular, a revolutionary iterative encryption is aimed to precisely preserve the spatial correlation of the original block in its encryption block, whereas a hybrid encoding aims to achieve high embedding capacity. High embedding capacity and security are the outcomes of this. Wu et al. [21] proposed a global sorting strategy combining local and global image features for reversible data hiding. For each pixel, its predicted value and local complexity are first calculated based on its local features. Then, according to the predicted value of the image pixels, the image pixels are globally sorted to generate a single sorted pixel sequence. Then, the sorting order of predictors based on location pixel value sorting is split to obtain a more regular two-dimensional histogram. Based on the generated regular histogram, they developed a more effective 2D mapping for data hiding. The experimental results show that the average PSNR after embedding 10,000 bits reaches 63.55 dB, which proves the superiority of the scheme.

### 1.3. Contribution of This Work

Driven by the above work and discussion, this paper proposes a *n*D-CTBCS, which can generate chaotic maps of arbitrary dimensions by using one-dimensional chaotic maps as seed chaotic maps. The performance of *n*D-CTBCS is discussed. To verify the validity of *n*D-CTBCS, three two-dimensional chaotic maps are generated using multiple one-dimensional chaotic maps as seed chaotic maps. Performance analysis shows that the new chaotic map has hyperchaotic behavior and a more uniformly distributed output. To illustrate the practicability of *n*D-CTBCS, this paper proposes a reversible data hiding scheme based on the newly generated chaotic mapping for the secure communication of medical images. The experimental results show that the proposed method has higher embedding capacity and higher security than the existing methods. The main contributions of this paper are as follows.

1. A simple and practical *n*-dimensional cosine-transform-based chaotic system (*n*D-CTBCS) chaotic coupling framework is proposed for generating arbitrary dimensional chaotic maps.
2. Apply multiple 1D chaotic maps to *n*D-CTBCS to generate three 2D chaotic maps. The performance is evaluated in theory and experiment, and the proposed chaotic map is compared with the most advanced chaotic map, showing excellent performance.
3. A reversible data hiding scheme is proposed for the secure communication of medical images, and the security analysis shows the remarkable performance of the scheme.

The rest of this article is organized as follows. Section 2 presents the introduction of the proposed *n*D-CTBCS, demonstrates several novel two-dimensional chaotic maps created using the *n*D-CTBCS, and evaluates the performance metrics of these newly generated chaotic maps. Section 3 gives a comprehensive description of the reversible data-hiding scheme of medical images based on the chaotic system. In Section 4, the performance of the proposed method is verified by experimental results and safety analysis. The last part is the summary of this paper.

## 2. *n*-Dimensional Chaotic Model

This section first presents the *n*-dimensional cosine-transform-based chaotic system (*n*D-CTBCS), then generates some new 2D chaos maps through *n*D-CTBCS based on some existing 1D chaos maps, and finally analyzes the performance of these new chaotic maps to show the advantages of the model.

### 2.1. nD-CTBCS

*n*D-CTBCS was created to address issues with chaotic systems that are currently in use. It takes *n* seed maps to generate *n*-dimensional chaotic systems, and the sum result of any two seed maps is performed by cosine transform, taking it as the output of the current dimension, iteratively replacing the seed maps of each dimension, and using the output of the current dimension as the input of the next dimension; *n*D-CTBCS is generated by combining the output of *n* dimensions. The mathematical structure of *n*D-CTBCS is defined as

(1) $$\left\{\begin{array}{c} x_{1,i+1}=cos\left(\pi \left(F_{1}\left(a_{1},x_{2,i}\right)\right)+F_{2}\left(a_{2},x_{2,i}\right)+\alpha_{1}\right)\;\;\;\;\;\;\;\;\;\;\;\;\;\;\;\;\;\;\;\;\;\;\;\;\;\;\;\\ x_{2,i+1}=cos\left(\pi \left(F_{2}\left(a_{2},x_{1,i+1}\right)\right)+F_{3}\left(a_{3},x_{3,i}\right)+\alpha_{2}\right)\;\;\;\;\;\;\;\;\;\;\;\;\;\;\;\;\;\;\;\;\;\;\;\\ x_{3,i+1}=cos\left(\pi \left(F_{3}\left(a_{3},x_{2,i+1}\right)\right)+F_{4}\left(a_{4},x_{4,i}\right)+\alpha_{3}\right)\;\;\;\;\;\;\;\;\;\;\;\;\;\;\;\;\;\;\;\;\;\;\;\\ \vdots \\ x_{n-1,i+1}=cos\left(\pi \left(F_{n-1}\left(a_{n-1},x_{n-2,i+1}\right)\right)+F_{n}\left(a_{n},x_{n,i}\right)+\alpha_{n-1}\right)\\ x_{n,i+1}=cos\left(\pi \left(F_{n}\left(a_{n},x_{n-1,i+1}\right)\right)+F_{1}\left(a_{1},x_{1,i}\right)+\alpha_{n}\right)\;\;\;\;\;\;\;\;\;\;\;\;\;\;\;\;\;\; \end{array}\right.,$$

where $F_{1}\left(a_{1}\right)$, $F_{2}\left(a_{2}\right)$, ⋯, $F_{n}\left(a_{n}\right)$ are n seed chaotic maps that are all 1D chaotic maps, and $a_{1},a_{2},\cdots a_{n}$ are the control parameter of the seed maps. $x\left(i\right)={\left\{x_{1,i},x_{2,i},\cdots ,x_{n,i}\right\}}^{T}$ is an n-length vector that is the ith observation state of the chaotic model and $\alpha_{1},\alpha_{2},\cdots \alpha_{n}$ are shifting constants. 

For any given parameter configuration, the cosine transform is a limited operation with complex nonlinearity that can produce chaotic occurrences. Consequently, the following traits of the suggested *n*D-CTBCS are present.

1. An efficient and straightforward chaotic generation model is the suggested *n*D-CTBCS model. By merging different seed chaotic maps, users can create chaotic maps in any dimension with flexibility. By switching the positions of the seed chaotic systems, several *n*D-CTBCS chaotic systems can be formed during the generation process.
2. The newly generated *n*D-CTBCS chaotic map can overcome the shortcomings of the existing chaotic interval discontinuity and uneven signal distribution.
3. $\alpha_{1},\alpha_{2},\cdots \alpha_{n}$ are introduced as the control parameters of the *n*D-CTBCS chaotic system to expand the parameter space, and the system can exhibit chaos in a large parameter range, while most existing chaotic systems only exhibit chaos in a very narrow parameter range.

### 2.2. Examples of 2D Chaotic Map

To demonstrate the advantages of *n*D-CTBCS in generating chaotic maps, we use *n*D-CTBCS to generate three 2D chaotic maps by using some chaotic maps as seed chaotic maps.

Firstly, four existing one-dimensional chaotic maps are introduced.

Logistic map (LM) is the most widely used nonlinear model of dynamic discrete chaotic systems [22], which is mathematically defined as

(2) $$x_{i+1}=\mu \;x_{i}\left(1-x_{i}\right),$$

where μ is the system parameter, μ∈[0,4]. 

Sine map [23], fractal map [2], and iterative chaotic map with infinite collapse (ICMIC) [24] are three common one-dimensional dynamic discrete chaotic mappings. The mathematical definition of the sine map is Equation (3).

(3) $$\;x_{i+1}=4sin\left(\pi x_{i}\right)/a,$$

where a is a system parameter. When a∈[0, 1], the mapping is in a chaotic state. The mathematical definition of a fractal map is Equation (4).

(4) $$x_{i+1}=1/x_{i}^{2}+0.1-bx_{i},$$

where b is the system parameter. When b∈[−0.999, 0.999], the mapping is in a chaotic state. ICMIC is defined as Equation (5).

(5) $$x_{i+1}=sin(c/x_{i}),$$

where c is the system parameter. When c∈(0, +∞), the mapping is in a chaotic state.

A bifurcation diagram is a tool to visualize the randomness of chaotic systems, and Lyapunov exponents (LE) are an important index to evaluate the chaotic identity of dynamic systems [1]. In this paper, the bifurcation diagram and Lyapunov exponent diagram of the above four one-dimensional chaotic maps are given.

**Figure 1:**
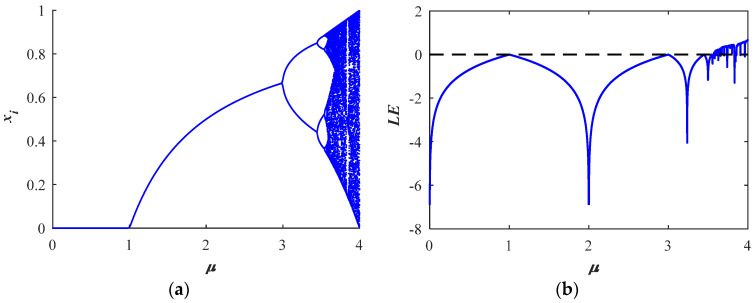

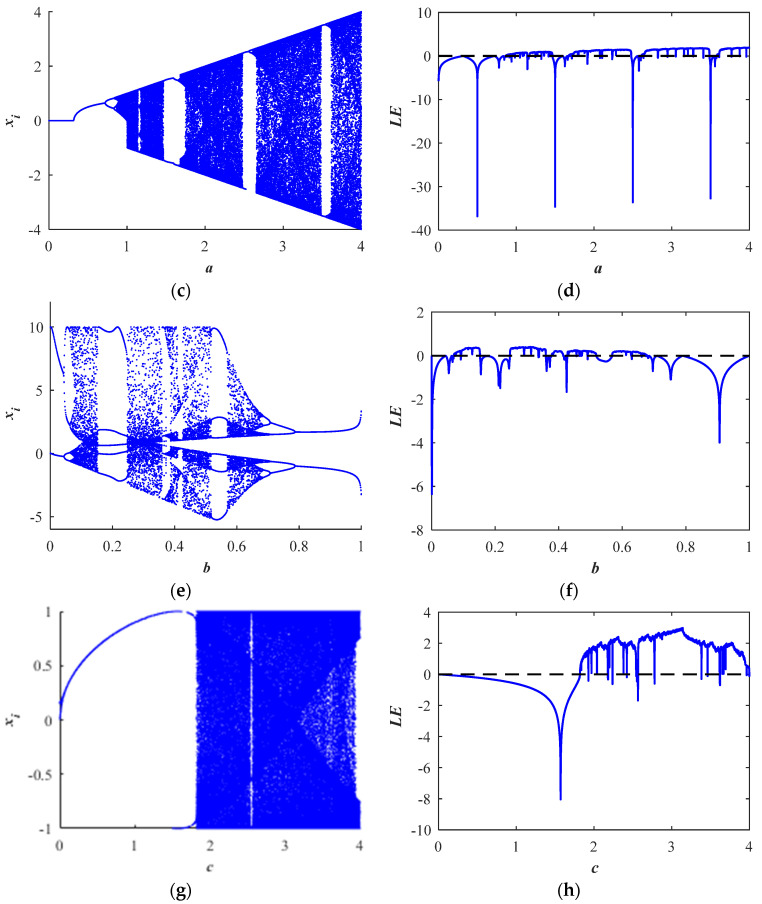
Bifurcation diagrams of (**a**) logistic; (**c**) sine; (**e**) fraction; (**g**) ICMIC maps; LEs of (**b**) logistic; (**d**) sine; (**f**) fraction; (**h**) ICMIC maps.

The first column of Figure 1 is the bifurcation diagram corresponding to the above four chaos diagrams. It can be seen that one-dimensional chaos mapping has defects such as narrow chaos range and period window. This means that the control parameters will not exhibit chaotic behavior beyond a certain interval. The second column of Figure 1 shows the Lyapunov exponents (LE) diagram corresponding to the above four chaotic mappings. It can be seen that the LEs of most one-dimensional chaotic systems are slightly greater than zero, but LE is still less than zero, which indicates that there is no chaos phenomenon under some parameters.

Therefore, this paper does not use the above four one-dimensional chaotic maps directly but uses them as the input of the coupled chaotic system proposed in this paper to construct an *n*-dimensional chaotic map with better chaotic properties.

#### 2.2.1. 2D Logistic–Sine Map

When logistic mapping and sine mapping are selected as the two seed chaotic mappings $F_{1}\left(\cdot \right)$ and $F_{2}\left(\cdot \right)$ in Equation (1), a new two-dimensional logistics–sine mapping (2D-LSM) is generated, whose mathematical equation is

(6) $$\left\{\begin{array}{c} x_{i+1}=cos\left(\pi \left(4/bsin(\pi x_{i})\right)+ay_{i}(1-y_{i})+\alpha_{1}\right)\\ y_{i+1}=cos\left(\pi \left(ax_{i+1}(1-x_{i+1})\right)+4/bsin(\pi y_{i})+\alpha_{2}\right) \end{array}\right.,$$

where a and b are the two control parameters of logistic mapping and sine mapping, respectively, and $\alpha_{1}$ and $\alpha_{2}$ are the newly introduced control parameters. Because the cosine transform is a bounded operation, the parameters a, b, $\alpha_{1}$, and $\alpha_{2}$ can have larger values.

#### 2.2.2. 2D Sine–ICMIC Map

When sine mapping and ICMIC are selected as the two seed chaotic mappings $F_{1}\left(\cdot \right)$ and $F_{2}\left(\cdot \right)$ in Equation (1), a new two-dimensional sine–ICMIC mapping (2D-SIM) is generated, whose mathematical equation is

(7) $$\left\{\begin{array}{c} x_{i+1}=cos\left(\pi \left(4/asin(\pi x_{i})\right)+sin(b/y_{i})+\alpha_{1}\right)\\ y_{i+1}=cos\left(\pi \left(sin(b/x_{i+1})\right)+4/asin(\pi y_{i})+\alpha_{2}\right) \end{array}\right.,$$

where a and b are the two control parameters of sine mapping and ICMIC, respectively, and $\alpha_{1}$ and $\alpha_{2}$ are the newly introduced control parameters. Because the cosine transform is a bounded operation, the parameters a, b, $\alpha_{1}$, and $\alpha_{2}$ can have larger values.

#### 2.2.3. 2D Sine–Fraction Map

When sine mapping and fraction mapping are selected as the two seed chaotic mappings $F_{1}\left(\cdot \right)$ and $F_{2}\left(\cdot \right)$ in Equation (1), a new two-dimensional sine–fraction mapping (2D-SFM) is generated, whose mathematical equation is

(8) $$\left\{\begin{array}{c} x_{i+1}=cos\left(\pi \left(4/asin(\pi x_{i})\right)+(1/y_{i}^{2}+0.1-by_{i})+\alpha_{1}\right)\\ y_{i+1}=cos\left(\pi \left(1/x_{i+1}^{2}+0.1-bx_{i+1}\right)+4/asin(\pi y_{i})+\alpha_{2}\right) \end{array}\right.,$$

where a and b are the two control parameters of sine mapping and fraction mapping, respectively, and $\alpha_{1}$ and $\alpha_{2}$ are the newly introduced control parameters. Because the cosine transform is a bounded operation, the parameters a, b, $\alpha_{1}$, and $\alpha_{2}$ can have larger values.

### 2.3. Performance Evaluations

To prove the advantages of 2D-LSM, 2D-SIM, and 2D-SFM, this paper uses a phase diagram, bifurcation diagram, Lyapunov exponents (LE), Permutation entropy (PE), and NIST SP800-22 test for verification.

#### 2.3.1. Phase Diagram

**Figure 2:**
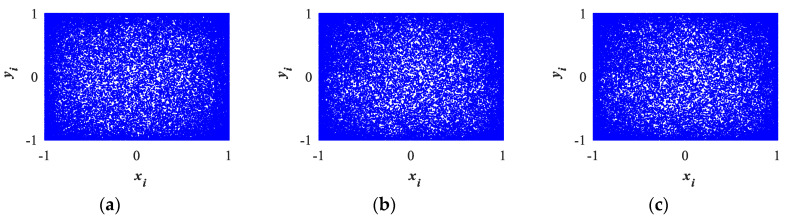

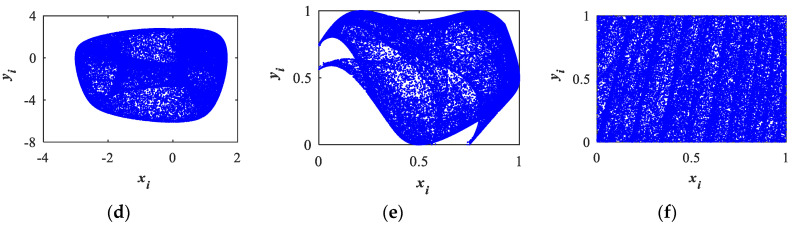
2D trajectories for different 2D chaotic maps: (**a**) 2D-LSM; (**b**) 2D-SIM; (**c**) 2D-SFM; (**d**) 2D-LSCM; (**e**) 2D-LSMCL; (**f**) 2D-LACM.

Plotting the approach and access points of a two-dimensional dynamic system with fixed parameter settings is performed using the phase space trajectory of a dynamic system [2]. This paper set up the initial parameters of 2D-LSM, 2D-SIM, and 2D-SFM as $x_{0}=0.1,y_{0}=0.1,a=0.03,b=0.04,\alpha_{1}=0.05,\alpha_{2}=0.06$; the two-dimensional phase space track as shown in Figure 2a–c.

Figure 2d–f are the 2D phase space trajectory diagrams of 2D-LSCM [25], 2D-LSMCL [26], and 2D-LACM [27], respectively. As can be seen from the figure, the distribution range of 2D-LSM, 2D-SIM, and 2D-SFM is significantly higher than that of 2D-LSCM and 2D-LSMCL. In addition, the distribution uniformity is better than that of 2D-LSCM, 2D-LSMCL, and 2D-LACM. This shows that the new system has superior ergodicity and randomness.

#### 2.3.2. Bifurcation Diagram

**Figure 3:**
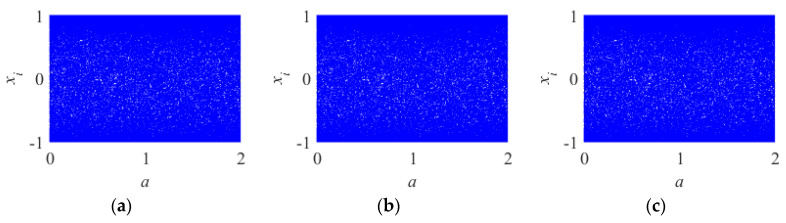
Bifurcation diagram for different 2D chaotic maps: (**a**) 2D-LSM; (**b**) 2D-SIM; (**c**) 2D-SFM.

A bifurcation diagram is a tool for visualizing the randomness of chaotic systems [28]. This paper set up the initial parameters of 2D-LSM, 2D-SIM, and 2D-SFM as $x_{0}=0.1,y_{0}=0.1,a=0.03,b=0.04,\alpha_{1}=0.05,\alpha_{2}=0.06$; their bifurcation distribution is shown in Figure 3.

As shown in Figure 3, the bifurcation distribution of 2D-LSM, 2D-SIM, and 2D-SFM does not have defects such as narrow range and period window. This means that chaotic behavior exists in all control parameter ranges.

#### 2.3.3. Lyapunov Exponents

The chaotic identity of dynamical systems can be assessed using Lyapunov exponents (LE) and maximum Lyapunov exponents (MLE), two crucial indices. The separation rate of very close trajectories is how LE defines chaos [2]. Mathematically, the following Equation (9) determines the LE of a dynamic system $D\left(x\right)$.

(9) $$LE=\underset{n\to \infty }{\lim}\left\{\frac{1}{n}\ln\left|\frac{D^{\left(n\right)}\left(x_{0}+\delta \right)-D^{\left(n\right)}\left(x_{0}\right)}{\delta }\right|\right\},$$

where δ represents a small positive value. For a dynamic system, its LE is equal to the dimension of its phase plane; a one-dimensional system has one LE, while a multidimensional system has several LEs. Positive Lyapunov exponents indicate a deviation from one-dimensional trajectories, suggesting that the dynamic system may exhibit chaotic behavior. The presence of two or more positive Lyapunov exponents indicates multi-dimensional divergence, potentially leading to hyperchaotic dynamics within the system. Furthermore, a larger positive LE denotes greater sensitivity to initial conditions, since LE characterizes the separation rate of extremely near orbits in chaotic systems [29].

**Figure 4:**
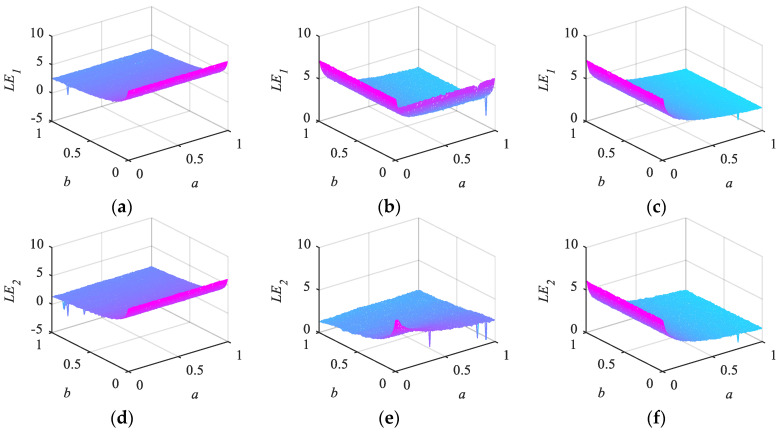
Two LEs for different 2D chaotic maps: (**a**) ${LE}_{1}$ of 2D-LSM; (**b**) ${LE}_{1}$ of 2D-SIM; (**c**) ${LE}_{1}$ of 2D-SFM; (**d**) ${LE}_{2}$ of 2D-LSM; (**e**) ${LE}_{2}$ of 2D-SIM; (**f**) ${LE}_{2}$ of 2D-SFM.

In this paper, the initial parameters of 2D-LSM, 2D-SIM, and 2D-SFM are set as $x_{0}=0.1,y_{0}=0.1,a=0.3,b=0.4,\alpha_{1}=0.5,\alpha_{2}=0.6$, and their two Lyapunov exponent distributions are shown in Figure 4. It can be seen that all three systems have two positive LEs, which can exhibit hyperchaotic behavior.

**Figure 5:**
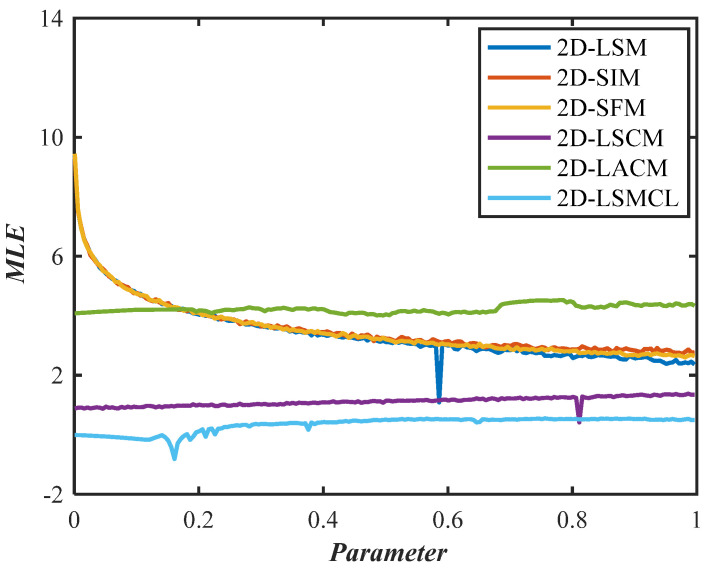
MLE of different chaotic maps.

In addition, the MLE distribution of 2D-LSM, 2D-SIM, 2D-SFM, 2D-LSCM, 2D-LSMCL, and 2D-LACM chaotic systems are shown in Figure 5. It is clear that the MLE values of 2D-LSM, 2D-SIM, and 2D-SFM have no significant window period and are higher than other chaos graphs. This means that the proposed mappings have more complex dynamic properties.

#### 2.3.4. Permutation Entropy

A technique for identifying dynamic mutation and randomness in time series is called permutation entropy (PE), which can be used to quantify random noise in signal sequences [30]. PE first creates a K-row matrix by reconstructing the time series. Next, a column index representing each element’s position is created by placing each reconstructed component in ascending order. This column index then creates a set of symbol sequences. Lastly, Equation (10) yields the PE of the time series.

(10) $$PE\left(m\right)=-\frac{\sum_{i=1}^{K}P_{i}\ln P_{i}}{\ln m!},$$

where $P_{i}$ represents the probability of obtaining the reconstructed component according to the symbol sequence, and m represents the embedding dimension.

**Figure 6:**
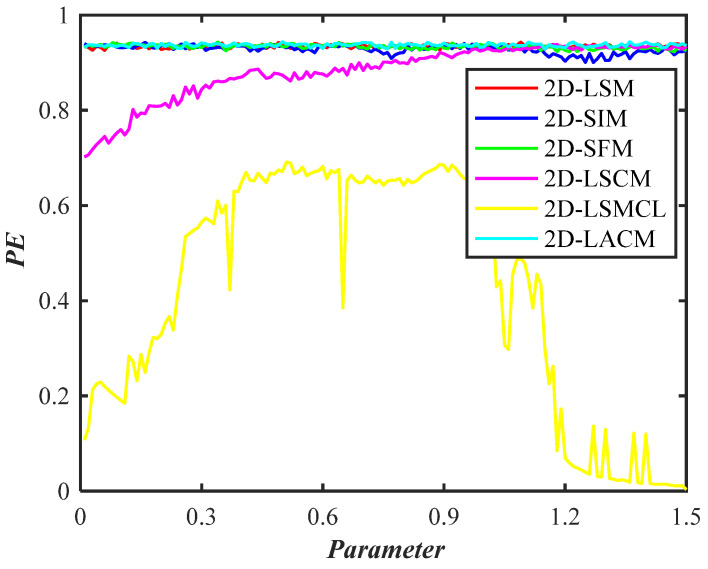
PEs of different chaotic maps.

PEs of different chaotic maps are shown in Figure 6. It can be seen that PEs of 2D-LSM, 2D-SIM, and 2D-SFM are comparable to those of 2D-LACM. Compared with other chaotic maps, PEs of 2D-LSM, 2D-SIM, and 2D-SFM are all larger and more stable. This shows that 2D-LSM, 2D-SIM, and 2D-SFM have better chaotic performance, and the generated sequences are more random and unpredictable.

#### 2.3.5. NIST SP800-22 Tests

The NIST SP800-22 tests contain 15 different tests and recommend $10^{3}$ to $10^{7}$ length sequences for testing [30]. This article uses $10^{6}$ length sequences. When the test value exceeds 0.01, the test sequence is random.

Table 1 shows results over 0.01, indicating that the six sequences produced by 2D-LSM, 2D-SIM, and 2D-SFM all pass the test and are random.

## 3. Reversible Data Hiding

Information security systems have made extensive use of chaotic systems due to their starting value sensitivity, unpredictability, ergodicity, and numerous other features. Shannon outlined the three fundamental information security systems—encryption, privacy, and hidden systems—in the Monograph on Information Security. This section describes the creation of a reversible data-hiding strategy for the secure transmission of stereoscopic medical images, based on 2D-LSM.

**Figure 7:**
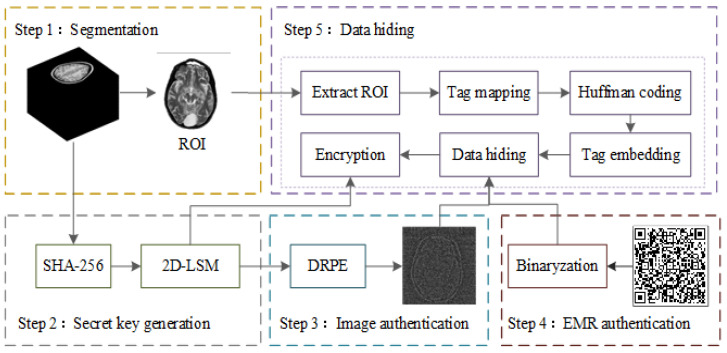
The flow chart of the proposed data-hiding algorithm.

As shown in Figure 7, the whole structure of the reversible data-hiding scheme based on 2D-LSM mainly consists of five stages: stereo image segmentation, key and chaotic sequence generation, image authentication, EMR authentication, and data hiding. Assuming the grayscale spiral CT image P is used as the object, these stages can be simply described as follows.

### 3.1. Stereo Image Segmentation

**Figure 8:**
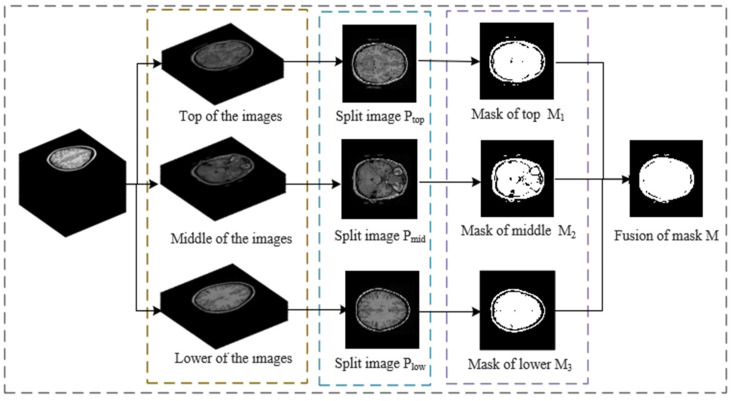
Segmentation mask generation process.

As shown in Figure 8, this section introduces the generation process of a stereoscopic image segmentation mask.

Step 1: Stereoscopic medical images are evenly divided into three parts: top, middle, and lower.

Step 2: The first image of these three parts was selected and denoted as $P_{top}$, $P_{mid}$, and $P_{low}$, respectively. The Otsu threshold segmentation method [7] was applied to segment $P_{top}$, $P_{mid}$, and $P_{low}$ to generate three segmentation masks, denoted as $M_{1},M_{2},M_{3}$.

Step 3: The three segmentation masks are added to obtain M, and the final segmentation mask M is generated according to Equation (11).

(11) $$M=\left\{\begin{array}{c} 1,(M_{1}+M_{2}+M_{3})>0\\ 0,(M_{1}+M_{2}+M_{3})\leq 0 \end{array}\right..$$

Step 4: The segmentation mask M was applied to the whole stereoscopic image and divided into a region of interest (ROI) and background region. The ROI was denoted as $P_{ROI}$, and pixels belonging to the background region were discarded.

### 3.2. Key and Chaotic Sequence Generation

The initial value and sequence generation steps of chaotic systems are as follows:

Step 1: The plaintext information $P_{top},P_{mid},P_{low}$ are taken as the input of the SHA-256 algorithm to obtain the hash values $K_{1},K_{2},K_{3}$, which are usually represented by a hexadecimal number of length 64.

(12) $$\left\{\begin{array}{c} K_{1}\left(k_{1},k_{2},\;k_{3}\ldots k_{64}\right)=SHA256(P_{top})\\ K_{2}\left(k_{1},k_{2},\;k_{3}\ldots k_{64}\right)=SHA256(P_{mid})\\ K_{3}\left(k_{1},k_{2},\;k_{3}\ldots k_{64}\right)=SHA256(P_{low}) \end{array}\right..$$

Step 2: Convert $K_{1},K_{2},K_{3}$ to 4-bit binary numbers, and then convert each group of eight to decimal numbers, and calculate the XOR according to the Equation (13) to generate a decimal array of length 32.

(13) $$\left\{\begin{array}{c} {key}_{1}\left({ka}_{1},{ka}_{2},\;{ka}_{3}\ldots {ka}_{32}\right)=K_{1}⊕K_{2}\\ {key}_{2}\left({kb}_{1},{kb}_{2},\;k_{b3}\ldots {kb}_{b32}\right)=K_{2}⊕K_{3} \end{array}\right..$$

Step 3: The initial parameters of the chaotic system are calculated according to Equation (14).

(14) $$\left\{\begin{array}{c} \begin{array}{c} x_{0}=({ka}_{1}⊕{ka}_{2}⊕\cdots ⊕{ka}_{16})/256\\ y_{0}=({ka}_{17}⊕{ka}_{18}⊕\cdots ⊕{ka}_{32})/256 \end{array}\\ \begin{array}{c} a={(kb}_{1}⊕{kb}_{2}⊕\cdots ⊕{kb}_{16})/256\\ b={(kb}_{17}⊕{kb}_{18}⊕\cdots ⊕{kb}_{32})/256 \end{array} \end{array}\right..$$

Step 4: Set the parameter c to 0.5 and the parameter d to 0.6, and substitute the calculated $x_{0},y_{0},a,b$ into the 2D-LSM hyperchaotic system; iteratively generate two random sequences, denoted x,y.

### 3.3. Image Authentication

Double random phase coding (DRPE) is an optical coding technique that is frequently used for picture authentication and encryption. It was first presented by Refregier et al. [31]. Equations (15) and (16) illustrate the double random phase encoding and decoding procedure.

(15) $${g(x,y)=FT}^{-1}\left\{FT\left\{f\left(x,y\right)exp\left[i2\pi \;\theta \left(x,y\right)\right]\right\}exp\left[i2\pi \;\varphi \left(u,v\right)\right]\right\},$$

(16) $$f\left(x,y\right)=\left\{{FT}^{-1}\left[FT\left(g\left(x,y\right)\right)\right]exp\left(-i2\pi \varphi \left(u,v\right)\right)\right\}exp\left(-i2\pi \theta \left(x,y\right)\right),$$

where f(x,y) is the original image, and g(x,y) is the encoded image. FT and ${FT}^{-1}$ represent the Fourier transform and the inverse Fourier transform, respectively. x and y are spatial coordinates, and u and v are frequency domain coordinates. θ(x,y) and φ(u,v) are two sets of two-dimensional normally distributed random numbers in the spatial domain and the frequency domain, whose values are randomly distributed between [0, 1], and the convolution and mean of the two arrays are zero; that is, they are two random white noises that are independent of each other. Therefore, exp(−i2πθ(x,y)) and exp[j2πφ(u,v)] are phase masks capable of producing phases between [0, 2π]. The encoding result g(x,y) is a complex amplitude with amplitude spectrum and phase spectrum. The phase spectrum of the encoding result is extracted as authentication information, and the correlation between the decoded image and the authenticated image can be verified by peak correlation energy.

Step 1: Calculate the size of the cover image $P_{top}$ according to Equation (17).

(17) $$\left[m,n\right]=Size(P_{top}).$$

Step 2: Two random phase plates were constructed, the first m×n bits of a random sequence x and y were intercepted, reconstructed according to Equation (18).

(18) $$\left\{\begin{array}{c} \theta \left(x,y\right)=\text{reshape}\left(x\left(1:m\times n\right),m,n\right)\\ \varphi (u,v)=\text{reshape}\left(y\left(1:m\times n\right),m,n\right) \end{array}\right..$$

Step 3: According to Equation (15), encoding results only retain the phase signal $P_{phase}$, and discard all amplitude information.

Step 4: According to Equation (19), the phase signal $P_{phase}$ is binarized.

(19) $$P_{phase}=im2bw\left(P_{phase}\right).$$

Step 5: $P_{phase}$ is converted into a one-dimensional sequence as the authentication information $A_{p}$ of the image.

### 3.4. EMR Authentication

Information security-related concerns will have an impact on the management of medical images. Three primary concerns are image source verification, whether the image matches the patient, and preventing the separation of the image from the associated electronic medical record (EMR) [7]. To prevent illegal copying and falsification of EMR data and to ensure that patient data and their corresponding medical images correspond, a QR code has been chosen as the container of the patient electronic medical record report in this paper. The QR code is embedded in stereoscopic medical image data.

Step 1: According to Equation (20), the QR code $P_{QR}$ is binarized.

(20) $$P_{QR}=im2bw\left(P_{QR}\right).$$

Step 2: $P_{QR}$ is converted into a one-dimensional sequence as the authentication information $A_{e}$ for EMR data.

### 3.5. Data Hiding

M, $A_{p},$ and $A_{e}$ as secret information are hidden in $P_{ROI}$.

Step 1: According to Algorithm 1, $P_{ROI}$ is divided into the embeddable region $P_{ROIe}$ and the unembeddable region $P_{ROIn}$.
**Algorithm 1** Embedded region partitioning algorithm**Input:**$\;\mathrm{Original}\;\mathrm{one}-\mathrm{dimensional}\;\mathrm{sequence}\;P_{ROI}$. **Output:**$\;\mathrm{Embeddable}\;\mathrm{matrices}\;P_{ROIe}$$\;\mathrm{and}\;\mathrm{non}-\mathrm{embeddable}\;\mathrm{sequences}\;P_{ROIn}$.1:$m,n\leftarrow size\left(P_{ROI}\right)$;2:long←m×n;3:squarelong←floor(sqrt(long));4:length←squarelong×squarelong;5:$P_{ROIe}\leftarrow P_{ROI}(1:length)$;6:$P_{ROIe}\leftarrow reshape(P_{ROIe},squarelong,squarelong)$;7:$P_{ROIn}\leftarrow P_{ROI}(length+1:end)$.

Step 2: The median edge detector (MED) predictor [32] was used to calculate the predicted value px(i,j) for each pixel x(i,j) of the embedded region $P_{ROIe}$.

Step 3: Convert the values of x(i,j) and px(i,j) into an 8-bit binary sequence denoted as xk(i,j) and pxk(i,j), where k=1,2,⋯,8.

Step 4: According to the method in Ref. [10], xk(i,j) and pxk(i,j) are compared successively from the most significant bit to the least significant bit until a certain bit is different, the label value of each pixel is recorded, and the theoretical embedding capacity of the entire image is calculated by adding all the label values. For these label values, Huffman coding is used for compression, reducing the amount of auxiliary information and increasing the payload of the image.

Step 5: According to Equation (21), auxiliary information such as Huffman coding rules and label mapping and secret information such as M, $A_{p}$, and $A_{e}$ are embedded into $P_{ROIe}$.

(21) $$P_{ROIe}^{\prime }=\left\{\begin{array}{c} P_{ROIe}\left(i,j\right)mod2^{7-t}+\sum_{s=0}^{t}\left(b_{s}\times 2^{7-s}\right),0\leq t\leq 6\\ \sum_{s=1}^{8}\left(b_{s}\times 2^{8-s}\right)\;\;\;\;\;\;\;\;\;\;\;\;\;\;\;\;\;\;\;\;\;\;\;\;\;\;\;\;\;\;\;\;\;\;\;\;\;\;,7\leq t\leq 8 \end{array}\right.,$$

where t is the label value of the current pixel, and $b_{s}$ is the secret information to be embedded.

Step 6: $P_{ROIe}^{\prime }$ is converted into a one-dimensional sequence and joined with $P_{ROIn}$, denoted as $P_{ROI}^{\prime }$.

Step 7: Equation (22) is used to process chaotic sequence x, and Equation (23) is used to process chaotic sequence y.

(22) $$X(i)=mod\left(floor\left(x\times 10^{15}\right),s\right)$$

(23) $$Y(i)=mod\left(floor\left(y\times 10^{15}\right),256\right)$$

where s represents the length of the sequence to be encrypted, i=1,2,⋯,s.

Step 8: Sort the sequence X(i), derive the index matrix V, all elements in V are non-repeatable integers ranging from 1 to s, and encrypt $P_{ROI}^{\prime }$ according to Equation (24).

(24) $$C_{ROI}(i)=P_{ROI}^{\prime }(V(i)⊕Y(V(i))⊕C_{ROI}(i-1)),$$

where $C_{ROI}\left(0\right)=0$,i=1,2,⋯,s, ⊕ represents the bit-level XOR operation [5].

At this point, the data-hiding and encryption process is complete.

## 4. Experiments for Simulation

In this section, a comprehensive evaluation of the proposed reversible data-hiding algorithm is conducted. The assessment encompasses multiple dimensions: visual security analysis, key space analysis, entropy analysis, histogram analysis, correlation analysis, embedding capacity, and the ratio of encoded pixels. All experiments were executed using MATLAB 2016b, running on a machine equipped with an Intel i5 processor and 16 GB of RAM.

### 4.1. Visual Security Analysis

**Figure 9:**
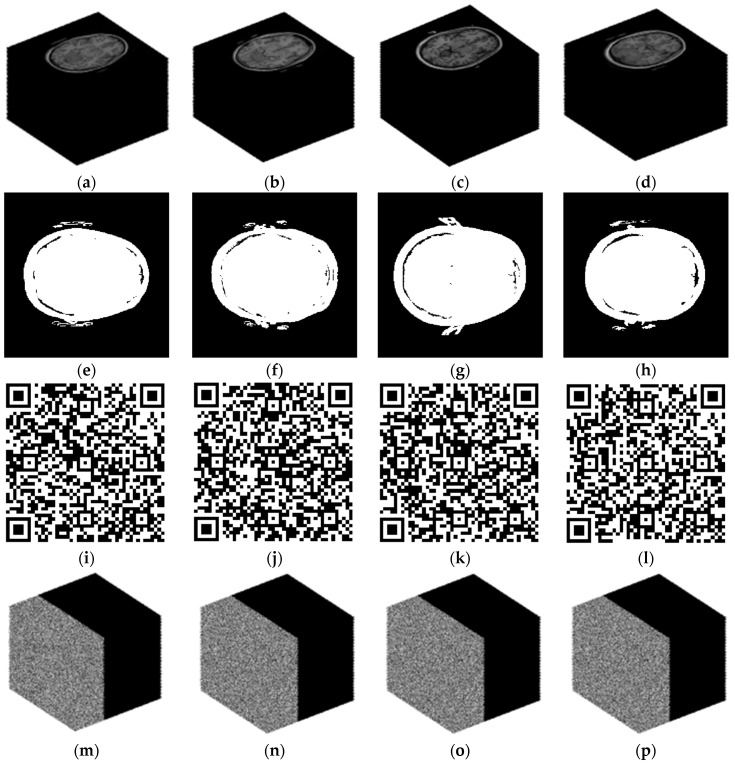
Reversible data-hiding results: (**a**) test1 image; (**b**) test2 image; (**c**) test3 image; (**d**) test4 image; (**e**) Mask of test1; (**f**) Mask of test2; (**g**) Mask of test3; (**h**) Mask of test4; (**i**) QR of test1; (**j**) QR of test2; (**k**) QR of test3; (**l**) QR of test4; (**m**) result of test1; (**n**) result of test2; (**o**) result of test3; (**p**) result of test4.

To validate the suggested reversible concealment technique, four sets of stereoscopic medical images are chosen from the TCIA dataset and used in this work. The four sets of images are named ${test}_{1}$, ${test}_{2}$, ${test}_{3}$, and ${test}_{4}$. The original stereo image of the four test image groups is shown in Figure 9a–d, the segmentation mask for the four test image groups is shown in Figure 9e–h, the QR code for the four test image groups is shown in Figure 9i–l, and the data-hiding results are shown in Figure 9m–p.

**Figure 10:**
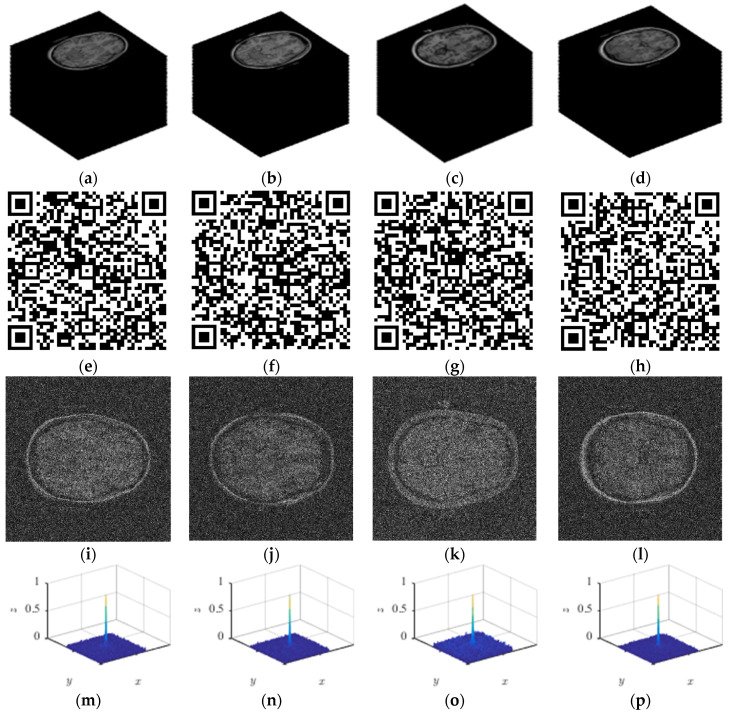
Decoding result: (**a**) test1 image; (**b**) test2 image; (**c**) test3 image; (**d**) test4 image; (**e**) QR of test1; (**f**) QR of test2; (**g**) QR of test3; (**h**) QR of test4; (**i**) authentication image of test1; (**j**) authentication image of test2; (**k**) authentication image of test3; (**l**) authentication image of test4; (**m**) PCE of test1; (**n**) PCE of test2; (**o**) PCE of test3; (**p**) PCE of test4.

The key point of reversible data hiding is to recover the original image and secret information without error. The qualitative analysis results of this algorithm are shown in Figure 10. The decoded stereoscopic image, QR code, and authentication image can be retrieved through reverse decoding.

To assess the reconstructed image quality quantitatively, the following metrics are introduced: the peak signal-to-noise ratio (PSNR) and the mean square error (MSE).

(25) $$MSE=\frac{1}{M\times N}\sum_{i=1}^{M}\sum_{j=1}^{N}{\left(X^{\prime }\left(i,j\right)-Y^{\prime }\left(i,j\right)\right)}^{2},$$

where M and N represent the size of the picture $X^{\prime }\left(i,j\right)$ and $Y^{\prime }\left(i,j\right)$.

(26) $$PSNR=10{log}_{10}\frac{{\left(2^{n}-1\right)}^{2}}{MSE},$$

where MSE stands for the mean square error as it appears in Equation (25). In order to quantify the correlation between decoded and authenticated images, peak-to-correlation energy (PCE) is introduced. The association between the decrypted image and the verified image is stronger the higher the PCE value [31].

(27) $$CO\left(x,y\right)=IFT\left({\left|P\left(\mu ,\eta \right)\cdot A\left(\xi ,\nu \right)\right|}^{k}\cdot e^{\varphi_{P}\left(\mu ,\eta \right)-\varphi_{A}\left(\xi ,\nu \right)}\right),$$

where P(μ,η) and A(ξ,ν) are the 2D Fourier transforms of the decoded image P and authentication image A; $\varphi_{P}\left(\mu ,\eta \right)$ and $\varphi_{A}(\xi ,\nu )$ are their phase images. The parameter k is usually set to 0.3.

As can be seen from Figure 10 and Table 2, the extracted secret information is the same as the original secret information, and the original image can be completely restored according to the extracted auxiliary information.

### 4.2. Key Space Analysis

To counter brute force attacks, the key space of the algorithm should be expanded as much as possible. When the key space exceeds $2^{100}$, the system has sufficient ability to resist violent attacks. The key for this work is generated by a hash algorithm, which is an algorithm that maps data of any length to a fixed-length string. SHA-256 is a hash family that generates a hash value of 256 bits in length, usually represented by 64 hexadecimal numbers.

Three images in the stereoscopic image are recorded as $P_{top}$, $P_{mid}$, and $P_{low}$, which are taken as the input of SHA-256 algorithm, and three hash values of 256 bits in length are generated as the key. Therefore, the key space of this work is $2^{256\times 3}=2^{768}$, much larger than $2^{100}$, and the key space is large enough to resist brute force attack.

### 4.3. Information Entropy Analysis

Shannon established the concept of information entropy, which may be used to reflect the randomness of information sources and describe the degree of information confusion [33]. When it comes to picture security, the better the secrecy and the worse the image recognition effect, the bigger the information entropy. Equation (28) is the information entropy formula.

(28) $$H\left(c\right)=-\sum_{i=0}^{2^{L}-1}P\left(c_{i}\right)\times {log}_{2}P\left(c_{i}\right),$$

where $P\left(c_{i}\right)$ is the statistical likelihood of having $c_{i}$ in a medical image, and $c_{i}$ is the ith gray quantity in an image. L stands for the gray levels, and an optimal entropy value is eight.

Table 3 reveals that the entropy of the original image is below 7.1. However, the entropy of the encoded image closely approximates the theoretical maximum of 8. As a result, steganographic and encrypted images are very random, making it harder for adversaries to extract useful information from them. The information entropy of this approach is larger, as can be seen from the comparative findings in Table 3, suggesting that this algorithm has more unpredictability and security.

### 4.4. Histogram Analysis

The frequency of each gray-level pixel in the image is described by the histogram [34]. After several images are encrypted, the histogram distribution of the encrypted image should be relatively similar to prevent an attacker from deriving any crucial information from the floating histogram of the encrypted image. The histogram cannot offer suggestions for statistical analysis when the difference between the histograms is not significant.

**Figure 11:**
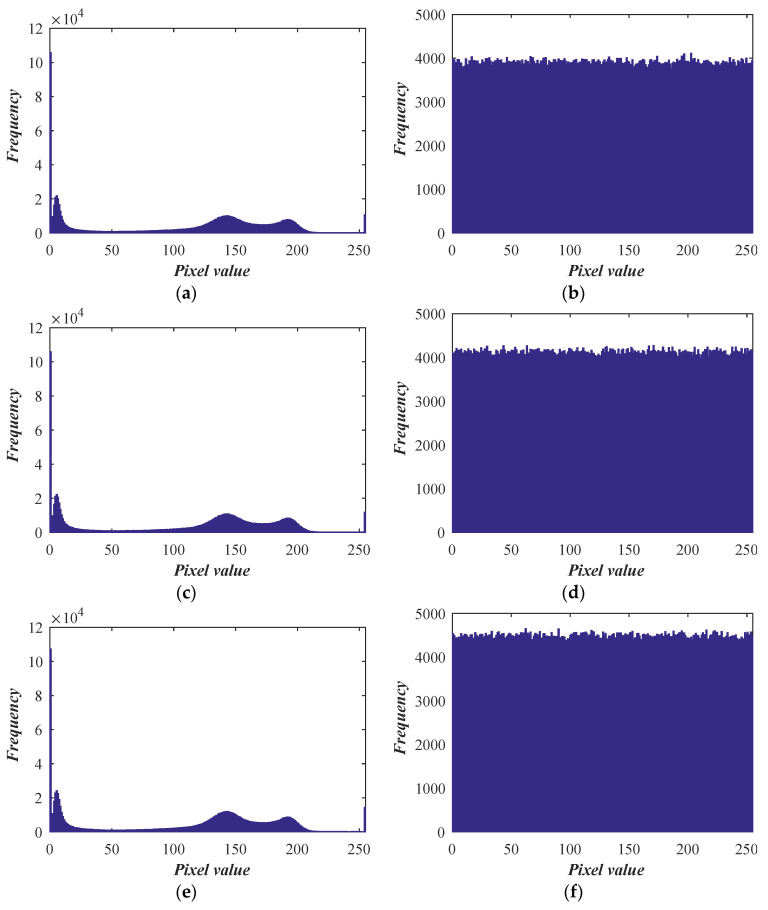

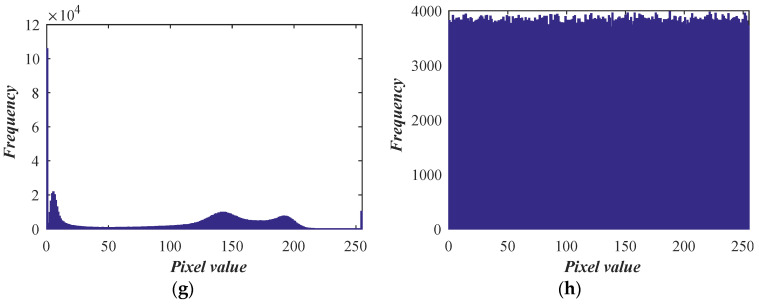
Histogram analysis: (**a**) histogram of test1; (**b**) histogram of encoded test1; (**c**) histogram of test2; (**d**) histogram of encoded test2; (**e**) histogram of test3; (**f**) histogram of encoded test3; (**g**) histogram of test4; (**h**) histogram of encoded test4.

The original image’s histogram, as seen in Figure 11, displays clear peaks and troughs that indicate the image’s statistical analysis characteristics. The uniform distribution of pixel values in the encoded image, on the other hand, significantly lowers the statistical correlation between the pixels among the features and has the benefit of fending off the statistical analysis’s assault.

The homogeneity of the histogram is statistically assessed in this research using the chi-square test. The more uniform the distribution of the encoded image, the smaller its chi-square test value. The method of measuring chi-squares is

(29) $$\chi^{2}=256\times \frac{\sum_{1}^{256}{\left(fp-\frac{M\times N}{256}\right)}^{2}}{M\times N},$$

where M and N represent the image size, and fp represents the number of gray values counted by the histogram.

The original image and the encoded image’s Chi-square test results are displayed in Table 4. The encoded image’s estimated result is significantly less than 1000, suggesting that the pixel frequency distribution is nearly uniform.

### 4.5. Correlation Analysis

The correlation coefficient between neighboring pair pixels can be formulated as

(30) $$\gamma_{xy}=\frac{cov\left(x,y\right)}{\sqrt{D\left(y\right)}\sqrt{D\left(x\right)}},$$

where $cov\left(x,y\right)=\frac{1}{K}\sum_{i=1}^{K}\left(x_{i}-E\left(x\right)\right)\left(y_{i}-E\left(y\right)\right)$, $D\left(x\right)=\frac{1}{K}\sum_{i=1}^{K}{\left(x_{i}-E\left(x\right)\right)}^{2}$, $E\left(x\right)=\frac{1}{K}\sum_{i=1}^{K}x_{i}$. K is the number of pixel pairs $\left(x_{i},y_{i}\right)$, $x_{i}$ and $y_{i}$ are the numerical values of two neighboring pair pixels, $E\left(x\right)$ and $E\left(y\right)$ are the mean values of the two adjoining pair pixels.

Table 5 displays the correlation coefficients’ quantitative analysis results between the original and encoded images. The encoded image’s correlation coefficients are close to 0 in all directions, while the original image’s correlation coefficients are almost 1 in all directions. A total of 2000 randomly chosen pixels are used in this paper’s qualitative correlation analysis.

**Figure 12:**
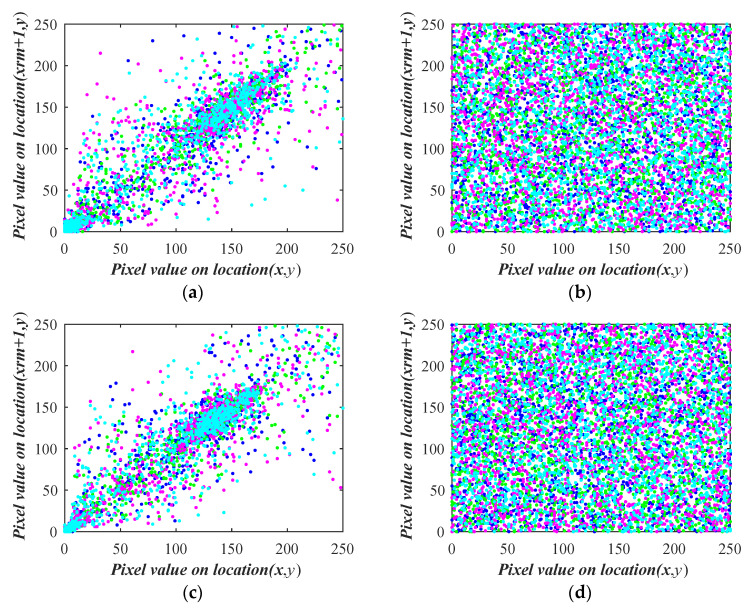

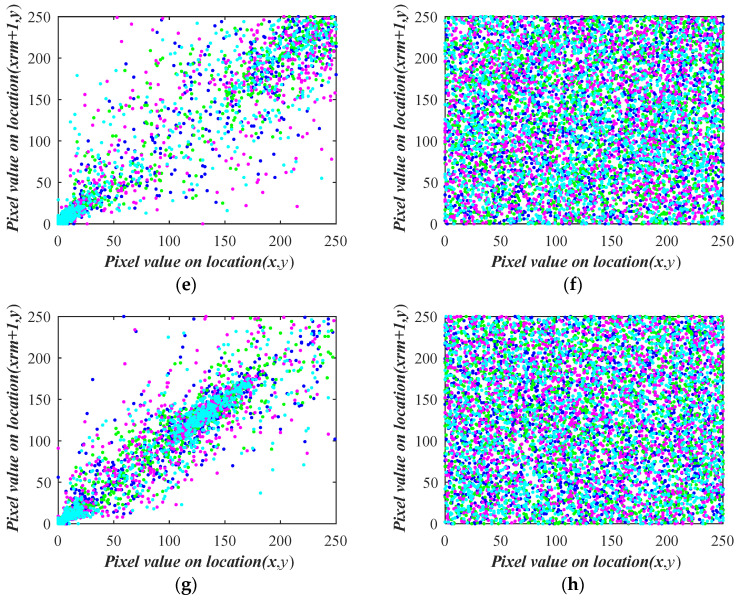
Correlation analysis: (**a**) test1-HVPS; (**b**) encoded test1-HVPS; (**c**) test2-HVPS; (**d**) encoded test2-HVPS; (**e**) test3-HVPS; (**f**) encoded test3-HVPS; (**g**) test4-HVPS; (**h**) encoded test4-HVPS.

The four colors in Figure 12 correspond to the four directions: horizontal (H), vertical (V), positive diagonal (P), and sub-diagonal diagonal (S). The correlation scatter plots in these directions are displayed. The coded image’s pixels are almost uniformly dispersed, whereas the plaintext image’s are grouped close to the diagonal. It suggests that the plan is resilient to statistical assaults.

### 4.6. Embedded Capacity Analysis

The image’s label map is computed using the MED predictor to provide an accurate prediction value. Huffman coding is used to decrease the auxiliary information, compress the label map, and increase the image’s payload to increase the payload. Conversely, employing the MED predictor can also efficiently decrease the number of reference pixels and raise the number of embedded pixels, thereby enhancing embedded rates (ER). Calculating the image’s total embedded capacity (EC) is possible once the label mapping has been established. Similarly, the length of auxiliary information (AL) can also be computed to determine the size of the net payload using label mapping and Huffman coding principles.

Table 6 shows the payloads of the four sets of test images. Among them, ${ER}_{1}$ represents the theoretical embedding capacity, and ${ER}_{2}$ represents the embedding capacity after embedding auxiliary information. It can be seen that the amount of auxiliary information is still relatively large, resulting in the actual embedding capacity as not very ideal.

### 4.7. Encoded Pixel Ratio Analysis

**Figure 13:**
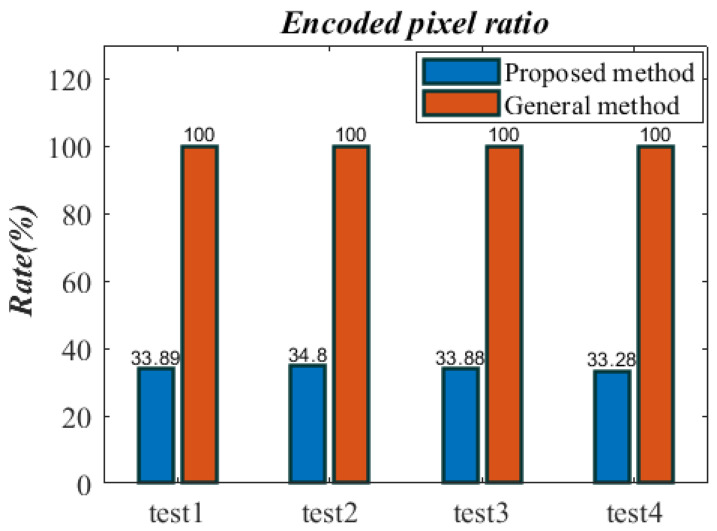
Encoded pixel ratio.

This work proposes a selective data concealing and encryption technique that eschews a significant amount of superfluous pixels. Less than 35% of the pixels in this research are encoded, as Figure 13 illustrates. This significantly lowers the computational load and boosts algorithm efficiency.

Compared with Ref. [7], the pixel encryption ratio in this paper is lower, and the amount of auxiliary information is reduced using fusion and segmentation mask, and the auxiliary information is embedded in the original image, and a complete steganographic encryption algorithm is constructed, which does not require additional space to store auxiliary information.

## 5. Conclusions

This research proposes an n-dimensional CTBCS (*n*D-CTBCS) chaotic coupling framework that is straightforward and useful for creating chaotic maps of any dimensionality. Three 2D chaotic maps are produced by applying many 1D chaotic maps to *n*D-CTBCS to illustrate its impact. We compare the proposed chaotic maps with the state-of-the-art chaotic maps. Based on the results, it can be concluded that all of the recently created chaotic maps perform better and display hyperchaotic behavior with broader chaotic intervals. To demonstrate the feasibility of *n*D-CTBCS, a reversible data hiding strategy is proposed to facilitate secure medical image communication. The stereoscopic image is divided into regions of interest and regions of disinterest, and the algorithm strategy is performed only on regions of interest, which reduces the number of encrypted pixels and does not require additional space to store auxiliary information. The results show that this method can be safely applied in the field of information transmission. Future work will focus on improving the practical embedding capabilities of reversible data hiding algorithms.

## Figures and Tables

**Table 1:** NIST SP800-22 test results for random number sequences.

NIST Test Items	2D-LSM		2D-SIM		2D-SFM	
Monobit frequency test	0.3859	0.1063	0.1181	0.3708	0.4231	0.5399
Frequency within block test	0.0865	0.7964	0.8398	0.8273	0.8312	0.9453
Runs test	0.5463	0.5732	0.9318	0.0129	0.1297	0.3399
Longest-run-ones in a block test	0.1988	0.1868	0.9880	0.1327	0.1808	0.9067
Binary matrix rank test	0.0371	0.2693	0.1616	0.0271	0.0352	0.0408
Discrete Fourier transform test	0.0422	0.1313	0.7496	0.7277	0.1240	0.5617
Non-overlapping template matching test	0.7323	0.6000	0.0562	0.2462	0.9697	0.3925
Overlapping template matching	0.9628	0.7664	0.0140	0.5182	0.0748	0.6554
Maurer’s universal statistical test	0.0536	0.4385	0.6654	0.8898	0.1186	0.3053
Linear complexity test	0.7108	0.1199	0.7434	0.3913	0.1876	0.5400
Serial test	0.1211	0.0544	0.4558	0.1086	0.0956	0.5779
Approximate entropy test	0.4136	0.0268	0.7791	0.6569	0.6032	0.3886
Cumulative sums test	0.0977	0.9716	0.9838	0.4371	0.9074	0.2756
Random excursion test	0.6382	0.6945	0.5854	0.6647	0.6414	0.6415
Random excursion variant test	0.4871	0.4065	0.5606	0.6500	0.3938	0.5142

**Table 2:** Quantitative analysis of image quality.

Image	MSE	PSNR	PCE
test1	0.0000	Inf	0.026538
test2	0.0000	Inf	0.026686
test3	0.0000	Inf	0.010429
test4	0.0000	Inf	0.030029

**Table 3:** Entropy analysis.

Image	Original	Encoded	Decoded
test1	7.0260	7.9998	7.0260
test2	7.0551	7.9998	7.0551
test3	7.0912	7.9999	7.0912
test4	7.0134	7.9999	7.0134
Ref. [33]	-	7.9993	-

**Table 4:** Histogram chi-square test.

Image	Original	Encoded	Decoded
test1	3.5545 × 10^6^	264.6750	3.5545 × 10^6^
test2	3.4451 × 10^6^	238.8966	3.4451 × 10^6^
test3	3.3803 × 10^6^	235.7004	3.3803 × 10^6^
test4	3.6059 × 10^6^	242.6068	3.6059 × 10^6^
Ref. [34]	1.3506 × 10^7^	262.5808	-

**Table 5:** Correlation analysis.

Image	H	V	P	S
test1-plain	0.9622	0.9724	0.9498	0.9365
test1-encoded	0.0483	−0.0020	0.0022	0.0112
test2-plain	0.9600	0.9792	0.9539	0.9573
test2- encoded	0.0060	−0.0013	−0.0088	−0.0076
test3-plain	0.9694	0.9761	0.9418	0.9507
test3- encoded	−0.0048	−0.0038	−0.0128	−0.0063
test4-plain	0.9646	0.9832	0.9545	0.9531
test4- encoded	0.0107	0.0061	0.0093	0.0048
Ref. [33]	0.0066	−0.0049	0.0158	-

**Table 6:** Embedded capacity analysis.

Image	Total EC (bits)	AL (bits)	Net Payload (bits)	${ER}_{1}\;(bpp)$	${ER}_{2}\;(bpp)$
test1	3985339	3209151	773743	3.9933	0.77774
test2	4119227	3387921	731306	3.9055	0.69336
test3	4395109	3665554	729555	3.8389	0.63722
test4	3925913	3151256	774657	4.0056	0.79039

## Data Availability

The used datasets are openly available from http://sipi.usc.edu/database/ and https://www.cancerimagingarchive.net/. The data used to support the findings of this study are available from the corresponding author upon reasonable request.

## Abbreviations

*n*D-CTBCS	*n*-dimensional cosine-transform-based chaotic system
1-DCMA	One-dimensional chaotic mapping amplifier
DH	Data hiding
RDH	Reversible data hiding
RDWT	Redundant discrete wavelet transform
NSST	Non-subsampled shear wave transform
LM	Logistic map
ICMIC	Iterative chaotic map with infinite collapse
LE	Lyapunov exponents
MLE	Maximum Lyapunov exponent
PE	Permutation entropy
2D-LSM	Two-dimensional Logistics–sine mapping
2D-SFM	Two-dimensional Sine–fraction mapping
2D-SIM	Two-dimensional Sine–ICMIC mapping
2D-LSCM	Two-dimensional Logistic–Sine–Cosine map
2D-LSMCL	Two-dimensional Logistic-modulated–Sine-coupling–Logistic chaotic map
2D-LACM	Two-dimensional Logistic-Adjusted-Chebyshev map
ROI	Region of interest
DRPE	Double random phase coding
EMR	Electronic medical record
MED	Median edge detector
PSNR	Peak signal-to-noise ratio
MSE	Mean square error
PCE	Peak-to-correlation energy
ER	Embedded rates
EC	Embedded capacity
AL	Auxiliary information
